# Medical service pricing and pharmaceutical supply chain coordination contracts under the zero-markup drug policy

**DOI:** 10.3389/fpubh.2023.1208994

**Published:** 2023-10-19

**Authors:** Nan Zhou, Shiyang Li, Gang Zhao, Chengjiang Li, Na Yu

**Affiliations:** ^1^College of Finance and Economics, Yangtze Normal University, Chongqing, China; ^2^College of Modern Postal, Chongqing University of Posts and Telecommunications, Chongqing, China; ^3^School of Engineering, University of Tasmania, Hobart, TAS, Australia; ^4^School of Management, Guizhou University, Guiyang, China; ^5^Shandong Provincial Hospital, Jinan, China

**Keywords:** pharmaceutical supply chain, medical service pricing, public benefit of hospital, coordination contracts, supply chain management

## Abstract

To implement state policies of zero-markup drug policy and medical service fee adjustment for public hospitals, this study constructed game models of the pharmaceutical supply chain, consisting of a drug supplier and a public hospital. The study obtained the optimal medical service level and pricing under the new state drug policies. In addition, it analyzed the impacts of the degree of public benefit of hospitals on the medical service level, the medical service price, and the drug price. Finally, from the perspective of cooperation between drug suppliers and public hospitals, the specialized coordination contract was designed to maximize overall social welfare. This study found an anomalous but meaningful conclusion: in the background of the zero-markup drug policy, a higher public benefit of hospitals could increase the drug prices, but it could reduce the medical service prices further to cut down on the overall treatment fees for the patients. The novel coordination contract can optimize the pharmaceutical supply chain and achieve a win-win situation for the drug suppliers, public hospitals, and patients. When the public benefit of hospitals is higher, the profit of a decentralized decision-making supply chain is greater than a centralized one, while the pharmaceutical supply chain will not coordinate itself.

## 1. Introduction

It is difficult and expensive to receive proper medical treatment in China, which has become one of the most significant domestic issues (1, 2). Under the policy of compensating medical service costs from drug sales profits, the drug markup policy is considered the main cause of expensive medical treatment (2). To break this vicious cycle, the state government working report declared that the drug markup policy, which compensates medical service costs with drug sale profits, must be abolished. Public hospitals had executed the policy nationwide by the end of December 2017. However, the income from drug sales is a major part of the revenues from public hospitals. It could adversely affect the operation of public hospitals when the government calls for the abolition of the drug markup policy to implement a drug zero-plus policy (3, 4). To solve this problem and compensate for the medical service costs provided by the public hospitals, the government implemented a healthcare system reform to adjust the medical service prices after implementing the drug zero-plus policy (5, 6). Since most public hospitals are state-owned public benefit organizations, it is important to embody their public benefit character during medical service pricing. However, there is no salient standard for the charging methods, charging levels, or medical service costs (7, 8). When medical services are priced too high, the total treatment cost increases even higher, and the character of public benefit is compromised. On the other hand, the basic operation costs cannot be covered if the services are priced too low when the drug markups are canceled. Some public hospitals asked the drug suppliers to reprice their products, disturbing the drug pricing system and intensifying the contradictions within the pharmaceutical supply chain (9). Therefore, fairly pricing medical treatment services has become a conundrum faced by public hospitals after implementing the drug zero-plus policy. Meanwhile, the drug zero-plus policy reconstructs the new relationship between the pharmaceutical supply chain members and forms a new profit distribution net. It has been the key to the success of the new policy to design a rational contract mechanism to increase pharmaceutical supply chain efficiency and strengthen its internal coordination.

With the background of China’s zero-markup drug policy and considering the public benefit characteristics of hospitals, a theoretical model of the pharmaceutical supply chain is established in this study. The medical service level and service price as well as drug prices from suppliers are analyzed under the new policy. Then, from the perspective of cooperation between the hospital and drug supplier, a coordination contract is proposed to reach a win-win situation for all the pharmaceutical supply chain members and for social welfare. Moreover, the implementation of the coordination contract is comprehensively discussed in the background of the new policy.

The rest of this study is arranged as follows: Section 2 is the literature review. Section 3 states the problem definition and hypothesis. Then, Section 4 compares equilibrium results between centralized and decentralized scenarios to explore the reason for supply chain inefficiency under a decentralized scenario. Section 5 proposes coordination contracts to maximize the overall social welfare. Section 6 gives the conclusion and significance of this study.

## 2. Literature review

Our study relates to two distinct areas of the existing literature: medical service pricing and pharmaceutical supply chain coordination contracts.

### 2.1. Medical service pricing

Due to the drug markup policy, the price of the medical service is rigorously controlled by the regulatory authorities, and its value has been underestimated in the last several decades (10). Wang et al. (11) analyzed the medical service pricing strategies in 30 provinces and found some problems in the charging range, standards, and medical insurance reimbursements. They suggested dynamic pricing and value correction for the medical service. Wang et al. (12) compared the pricing system between Shanghai and the international conventional one and suggested Shanghai use the standard price model to adjust based on the local price parameters. Many Western countries separate medical services from drug sales while reasonably pricing medical services. Some studies focused on pricing methods such as the cost-oriented pricing method (13), the cost-oriented pricing method (14), and the peer pricing-oriented method (15). Some other studies constructed the game models to explore the pricing strategies and balance under the competitive environment. Duan et al. (16) constructed a bargaining game model between service providers, government, and patients and investigated adverse selection in a diagnosis-related group system. Allen and Gertler (17) adopted the game theoretical model to discuss the satisfaction of the patients with the pricing strategies. Robinson (18) also used the game theoretical model to find the relationship between cost shift and payment. Some other researchers focused on the factors that influence medical service pricing, including government interventions (17, 19), medical insurance payment and ratio (20, 21), service quality (22, 23), etc. The differences between this study and the above research are mainly in two ways. First, there are few studies that consider the impact of the public benefit of hospitals on the pricing of medical services. Second, no literature has been found to consider the game relationships between members of the pharmaceutical supply chain in the pricing of medical services.

### 2.2. Pharmaceutical supply chain coordination contracts

In addition to the pricing of the medical services, the pharmaceutical supply chain coordination contract is another major topic in this study. Some previous studies (24–30) explored the influences of information asymmetry, risk aversion, demand uncertainty, cap-and-trade regulation, contract sequence, and cutoff policies to design coordination contract mechanisms to resolve conflicts within the supply chain. Some other researchers focused on specific products to design coordination contract mechanisms. For example, Gao et al. (31) focused on green products, Moon et al. (32) aimed at agricultural products, and Heydari et al. (33) specifically focused on remanufacturing products. Although the aforementioned literature provides numerous coordination contracts to resolve supply chain conflicts, these contracts are not specific to the pharmaceutical supply chain. Only a small number of studies have investigated the pharmaceutical supply chain and its coordination contracts. Weraikat et al. (34) investigated the reverse recycling of the supply chain, which consists of manufacturers, logistics companies, and consumers. They found that suitable coordination mechanisms could ensure the recycling of the drug products. Nematollahi et al. (35) explored the two-level pharmaceutical supply chain of suppliers and retailers and obtained the Pareto optimal solutions using the ε-constraint method, considering the visiting time and safe inventory levels. Tat et al. (36) researched the supply chain of suppliers and stores, considering corporate social responsibility and uncertain demands. The revenue and cost-sharing contract could effectively stimulate social responsibility and reduce drug loss. Studies (34–36) examined the coordination strategies of different types of pharmaceutical supply chains. However, there are some gaps between their supply chains and the one in this study. On the one hand, the background of the pharmaceutical supply chain in this study is the zero drug markup, which is a unique characteristic, especially in the Chinese medical market. On the other hand, while the above coordination contract is based on profit maximization, this study takes into account the public-benefit characteristics of hospitals and designs the contract to maximize social welfare.

## 3. Problem definition and hypothesis

**Figure 1:**
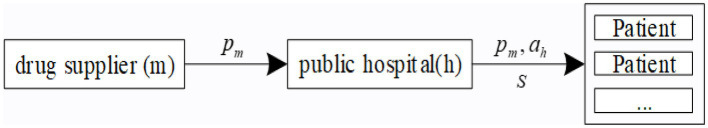
Pharmaceutical supply chain structure.

In the model of this study, the pharmaceutical supply chain consists of a drug supplier (m), a public hospital (h), and a group of patients. The drug supplier sells drugs to patients through a public hospital. Under the government’s zero-markup drug policy, the public hospital sells drugs to patients at the original prices $p_{m}$ provided by drug suppliers without adding any profit. The public hospital provides medical services s to patients. Meanwhile, the public hospital charges patients for their medical services at the price $a_{h}$ (Figure 1). It is generally assumed that some people suffering from a disease require medical services, and the total market demand is set as 1.

The patients’ individual utility as fully healthy is assumed to be u . In case of disease, the disease level of the patient is denoted by δ . There is a very wide variation in the level of disease in patients with the same disease; the disease level could vary significantly within different patients. For example, patients with the same type of hypertension can be categorized as low-risk, intermediate-risk, high-risk, or very high-risk as the value of their blood pressure increases. Without loss of generality, we assume that δ is a random value uniformly distributed between 0 and 1. Based on the assumptions above, the patient’s mental or physical damages caused by the disease is uδ , the retained utility of the patient is referred to as $U_{0}=u\left(1-\delta \right)$ . The public hospital provides the patient with medical services s and charges their medical services at the price $a_{h}$ . It is assumed that all patients who receive medical services from public hospitals will purchase drugs at a price $p_{m}$ . When patients receive treatment in a public hospital, they could obtain the utility of uδ , which means being recovered by the hospital. As a result, the patient’s utility in choosing a public hospital for treatment could be calculated by $U_{h}=U_{0}+u\delta -p_{m}-a_{h}+\eta s$ , where $\eta \in \left(0,\;1\right)$ refers to the coefficient of the patient’s preference for the medical services.

**Figure 2:**
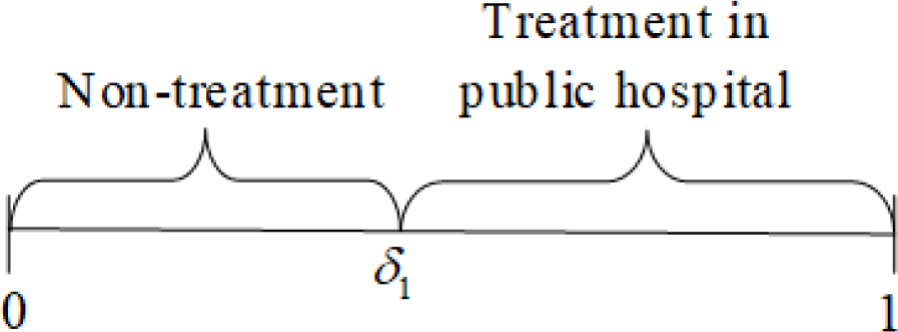
Patient treatment options.

As a rational consumer, patients could choose whether or not to receive treatment from the public hospital according to the principle of utility maximization, i.e., $U_{\text{max}}={\left(U_{0},U_{h}\right)}_{\text{max}}$ . When $U_{0}>U_{h}$ , the patient’s disease level satisfies $0<\delta <\delta_{1}$ , where $\delta_{1}=\frac{\left(p_{m}+a_{h}-\eta s\right)}{u}$ , the patient does not receive any medical treatment. When $U_{0}⩽U_{h}\text{,}$ the disease level satisfies $\delta_{1}\leq \delta <1$ , the patient chooses to receive medical treatment from a public hospital (Figure 2). Assuming the utility satisfies u=1 when the patient is fully healthy, the demand for choosing to receive treatment is expressed as $q_{h}=\overset{1}{\underset{p_{m}+a_{h}-\eta s}{\int }}1d\delta =1-\left(p_{m}+a_{h}-\eta s\right)$ .

Following the literature references (37, 38), the total utility of a public hospital is calculated by $v_{h}=\pi_{h}+\beta \cdot CS_{h}$ , where $\pi_{h}$ is the profit of the public hospital. *β* denotes the public benefit coefficient of the hospital, which satisfies *β* ∈ [0, 1]. The larger the coefficient *β* is, the higher the public benefit of the hospital will be. *β* = 0 indicates that the public hospital is a completely profit-driven institution. *β* = 1 indicates that the public hospital is a completely non-profit institution. $CS_{h}=\overset{1}{\underset{\delta_{1}}{\int }}\left(\delta -p_{m}-a_{h}+\eta s\right)d\delta$ refers to the total surplus number of the patient group who chooses the public hospital for treatment. Without loss of generality, the drug supplier’s acquisition cost is set as $c_{m}$ , and the fixed unit operation cost of a public hospital is set as $c_{h}$ (Table 1).

**Table 1:** Lists of the notations and symbols.

Notation	Description	Notation	Description
$\pi_{m}/\pi_{h}$	The profit of the drug supplier/the public hospital	β	The public benefit coefficient of the hospital
$v_{h}$	The total utility of public hospital	$CS_{h}$	The total surplus number of the patient group
u	The patients’ individual utility in fully healthy	δ	The disease level is denoted
$a_{h}$	The price of medical services	s	The medical service level of public hospital
$p_{m}$	The price of the drugs	η	The coefficient of the patient’s preference for medical services
$c_{m}$	The cost of drug supplier	$c_{h}$	The cost of public hospital

## 4. Modeling and analysis

In this section, the equilibrium solutions of the pharmaceutical supply chain under decentralized decision-making (indicated by superscript D) and centralized decision-making (indicated by superscript C) scenarios are delivered first. An analysis of the decision-making of public hospitals and drug suppliers is provided. The causes of pharmaceutical supply chain inefficiency are discussed by comparing two equilibrium solutions in two decision-making scenarios.

### 4.1. Decentralized decision-making

Public hospitals are usually in a dominant position in the Chinese domestic pharmaceutical supply chain. The public hospitals usually define the medical service level s and the service price *a_h_*, and then the drug supplier makes decisions on the sales price of drugs $p_{m}$ . The variable cost of the medical services level is assumed to be $C\left(s\right)$ , where $C\left(s\right)$ satisfies $\partial C\left(s\right)/\partial s>0$ and $\partial^{2}C\left(s\right)/\partial s^{2}>0$ . Without loss of generality, the variable cost of the medical services is assumed to be $C\left(s\right)=\frac{1}{2}s^{2}$ , and the decision strategies of public hospitals and drug suppliers can be expressed as the following equations:


(1) $$\max_{a_{h}s}v_{h}^{D}=\left(a_{h}-c_{h}\right)\cdot q_{h}+\beta \cdot \overset{1}{\underset{\delta_{1}}{\int }}\left(\delta -p_{m}-a_{h}+\eta s\right)d\delta -\frac{1}{2}s^{2}$$

(2) $$\max_{p_{m}}\pi_{m}^{D}=\left(p_{m}-c_{m}\right)\cdot q_{h}$$

Several theorems and propositions could be obtained by solving these equations via the backward induction method.

Theorem 1:

The equilibrium solutions of the pharmaceutical supply chain defined by the public hospital are:


$$p_{m}^{D*}=\frac{1-\eta^{2}c_{m}-\beta c_{m}-c_{h}+3c_{m}}{4-\eta^{2}-\beta }$$

$$a_{h}^{D*}=\frac{c_{h}\left(2-\eta^{2}\right)+\left(1-c_{m}\right)\left(2-\beta \right)}{4-\eta^{2}-\beta }$$

$$s^{D*}=\frac{\eta \left(1-c_{h}-c_{m}\right)}{4-\eta^{2}-\beta }$$

The equilibrium profit and utility of the public hospital are:


$$\pi_{h}^{D*}=\frac{{\left(1-c_{h}-c_{m}\right)}^{2}\left(4-\eta^{2}-2\beta \right)}{2{\left(4-\eta^{2}-\beta \right)}^{2}}$$

$$v_{h}^{D*}=\frac{{\left(1-c_{h}-c_{m}\right)}^{2}}{2\left(4-\eta^{2}-\beta \right)}$$

The equilibrium profit and patient surplus number of the drug supplier are:


$$\pi_{m}^{D*}=\frac{{\left(1-c_{h}-c_{m}\right)}^{2}}{{\left(4-\eta^{2}-\beta \right)}^{2}}$$

$$CS^{D*}=\frac{1}{2}\pi_{m}^{D*}$$

The profit and social welfare of the pharmaceutical supply chain are:


$$\pi_{SC}^{D*}=\frac{{\left(1-c_{h}-c_{m}\right)}^{2}\left(6-\eta^{2}-2\beta \right)}{2{\left(4-\beta -\eta^{2}\right)}^{2}}$$

$$SW^{D*}=\frac{{\left(1-c_{h}-c_{m}\right)}^{2}\left(7-\eta^{2}-2\beta \right)}{2{\left(4-\eta^{2}-\beta \right)}^{2}}$$

where $\pi_{SC}=\pi_{m}+\pi_{h}$ refers to the supply chain profit, CS is the surplus number of patients receiving treatment, and $SW=\pi_{m}+\pi_{h}+CS$ refers to the overall social welfare.

Proposition 1:


$$\left(1\right)\;\frac{\partial a_{h}^{D*}}{\partial \beta }<0,\frac{\partial s^{D*}}{\partial \beta }>0,\frac{\partial p_{m}^{D*}}{\partial \beta }>0,\left|\frac{\partial a_{h}^{D*}}{\partial \beta }\right|>\left|\frac{\partial p_{m}^{D*}}{\partial \beta }\right|$$

$$\left(2\right)\;\frac{\partial \pi_{h}^{D*}}{\partial \beta }<0,\frac{\partial v_{h}^{D*}}{\partial \beta }>0,\frac{\partial \pi_{m}^{D*}}{\partial \beta }>0,\frac{\partial CS^{D*}}{\partial \beta }>0$$

$$\left(3\right)\;\frac{\partial \pi_{SC}^{D*}}{\partial \beta }>0,\frac{\partial SW^{D*}}{\partial \beta }>0$$

Proposition 1 shows that the public benefit of hospitals is the main factor affecting the level and price of medical services. At a higher value of public benefit, the price of medical services is low while the price of drugs is high. Further study indicates that for each unit of public benefit from hospital improvement, the price of medical services decreases faster than the increase in drug prices. It means that the overall fee ( $a_{h}^{D*}+p_{m}^{D*}$ ) for patients to receive treatment at a public hospital decreases when the public benefit of the hospital increases. This suggests that only increasing the public welfare of hospitals can reduce overall treatment expenditures for patients, but this will excessively reduce the charges for medical services while the price of drugs will increase instead. Under the zero-markup drug policy, controlling the price of medical services by improving public welfare for medicines is effective, but curbing the price of medicines is not desirable.

A more interesting conclusion is that the profit of the public hospital ( $\pi_{h}^{D*}$ ) become less profitable but its total utility ( $v_{h}^{D*})$ increase with the increase of the public benefit. The main reason is that when the public benefit increases, the profit of the public hospital decreases, but the patient surplus increases. The increase in patient surplus number is greater than the profit loss of the public hospital, so the total utility of the public hospital increases.

From the supply chain perspective, the drug supplier’s profit increases, which is faster than the decrease in profit the public hospital suffers. Thus, the profits of the pharmaceutical supply chain increase as the public benefit of hospitals increases. At the same time, the patient surplus number increases as the public benefit of the hospital increases, which leads to an increase in social welfare. It shows that increasing the public benefit of hospitals will reduce hospital profits but benefit drug supplier profits, supply chain profits, and overall social welfare under the zero-markup drug policy. It is also a reminder that although improving the public welfare of hospitals benefits patients and society, it may be resisted by hospitals.

### 4.2. Centralized decision-making

Under centralized decision-making, the drug supplier’s drug sales are integrated with the medical services of the public hospitals. The pharmaceutical supply chain makes decisions to optimize system-wide profit. Under centralized decision-making, it is assumed that the overall fee provided to the patient is $p^{C}$ and the service provided is $s^{C}$ . According to the principle of utility maximization, when the patient’s disease level satisfies $0<\delta <\delta^{C}$ , where $\delta^{C}=p^{C}-\eta s^{C}$ , the patient does not receive any treatment; when the patient’s disease level satisfies $\delta^{C}\leq \delta <1$ , the patient receives treatment from the public hospital. Based on these principles, the patient’s demand is calculated by $q_{h}=\overset{1}{\underset{p^{C}-\eta s^{C}}{\int }}1d\delta =1-\left(p^{C}-\eta s^{C}\right)$ .

In this case, the pharmaceutical supply chain decision problem can be expressed as:


(3) $$\begin{array}{c} \max_{p^{C},s^{C}}v^{C}=\left(p^{C}-c_{m}-c_{h}\right)\left(1-\left(p^{C}-\eta s^{C}\right)\right)\\ \;+\beta \cdot \overset{1}{\underset{\delta_{c}}{\int }}\left(\delta -p^{C}+\eta s^{C}\right)d\delta -\frac{1}{2}{\left(s^{C}\right)}^{2} \end{array}$$

Theorem 2:

The optimal solution of the system under the centralized strategy is:


$$p^{C*}=\frac{\left(1-\eta^{2}\right)\left(c_{h}+c_{m}\right)-\beta +1}{2-\eta^{2}-\beta }$$

$$s^{C*}=\frac{\eta \left(1-c_{h}-c_{m}\right)}{2-\eta^{2}-\beta }$$

The profit, patient surplus number, and social welfare of the pharmaceutical supply chain are:


$$\pi_{SC}^{C*}=\frac{{\left(1-c_{h}-c_{m}\right)}^{2}\left(2-\eta^{2}-2\beta \right)}{2{\left(2-\eta^{2}-\beta \right)}^{2}}$$

$$CS^{C*}=\frac{{\left(1-c_{h}-c_{m}\right)}^{2}}{2{\left(2-\eta^{2}-\beta \right)}^{2}}$$

$$SW^{C*}=\frac{{\left(1-c_{h}-c_{m}\right)}^{2}\left(3-\eta^{2}-2\beta \right)}{2{\left(2-\eta^{2}-\beta \right)}^{2}}$$

Proposition 2:


$$\left(1\right)\;\frac{\partial p^{C*}}{\partial \beta }<0,\frac{\partial s^{C*}}{\partial \beta }>0.$$

$$\left(2\right)\;\frac{\partial \pi_{SC}^{C*}}{\partial \beta }<0,\frac{\partial CS^{C*}}{\partial \beta }>0,\frac{\partial SW^{C*}}{\partial \beta }>0$$

Proposition 2 shows that under a centralized decision-making scenario, with the increased public benefit of the pharmaceutical supply chain, patients pay less for disease treatment but receive a higher level of medical services instead. When the public benefit is high, the profit of the pharmaceutical supply chain is low, while the patient surplus number is high. The increase in the patient surplus number is greater than the decrease in supply chain profit, so the overall welfare of the pharmaceutical supply chain increases. It suggests that with the increase in public benefit, the pharmaceutical supply chain becomes less profitable, but patient surplus and overall welfare improve under a centralized decision-making scenario.

### 4.3. Comparison and analysis of two decision-making scenarios

After the study of the optimal pricing and performance of the pharmaceutical supply chain under decentralized and centralized decision-making scenarios, the specific characteristics of these two strategies will be compared and analyzed to support the contract coordination in this section.

Proposition 3:


$$\left(1\right)\;s^{D*}<s^{C*},\;p_{m}^{D*}+a_{h}^{D*}>p^{C*}$$

(2) when $0\leq \beta <\beta_{1}$ , there exists $\pi_{SC}^{D*}<\pi_{SC}^{C*}$ ; when $\beta_{1}\leq \beta \leq 1$ , there exists $\pi_{SC}^{D*}\geq \pi_{SC}^{C*}$ . $\mathrm{Of}\;\mathrm{which},\beta_{1}=2-\frac{1}{2}\eta^{2}-\frac{1}{2}\sqrt{\eta^{4}-4\eta^{2}+8}$ .


$$\left(3\right){\;SW}^{D*}<{SW}^{C*}\text{.}$$

This proposition implies that the level of medical services under decentralized decision-making is smaller than that under centralized decision-making, while the fee paid by the patient under decentralized decision-making is larger than the fee under centralized decision-making. As a result, social welfare is lower under decentralized decision-making than that under centralized decision-making.

Under decentralized decision-making, the drug supplier and the public hospital make decisions based on their priorities, i.e., the drug supplier pursues maximum profit, and the public hospital pursues maximum total utility. Due to the inconsistency of decision objectives and the double marginalization caused by the two decisions, the pharmaceutical supply chain deviates from the overall optimization. As a result, the quality of medical service patients receive reduces, and the overall fee of medical treatment increases, leading to a reduction in the overall welfare level.

From the perspective of the pharmaceutical supply chain, when the public benefit coefficient satisfies $0\leq \beta <\beta_{1}$ , the pharmaceutical supply chain profit under decentralized decision-making is smaller than the centralized one. When the public benefit coefficient satisfies $\beta_{1}\leq \beta \leq 1$ , the pharmaceutical supply chain profit under decentralized decision-making is larger than the centralized one. With the increase in the public benefit of hospitals, the supply chain profit increases under the decentralized decision and decreases under the centralized one.

**Figure 3:**
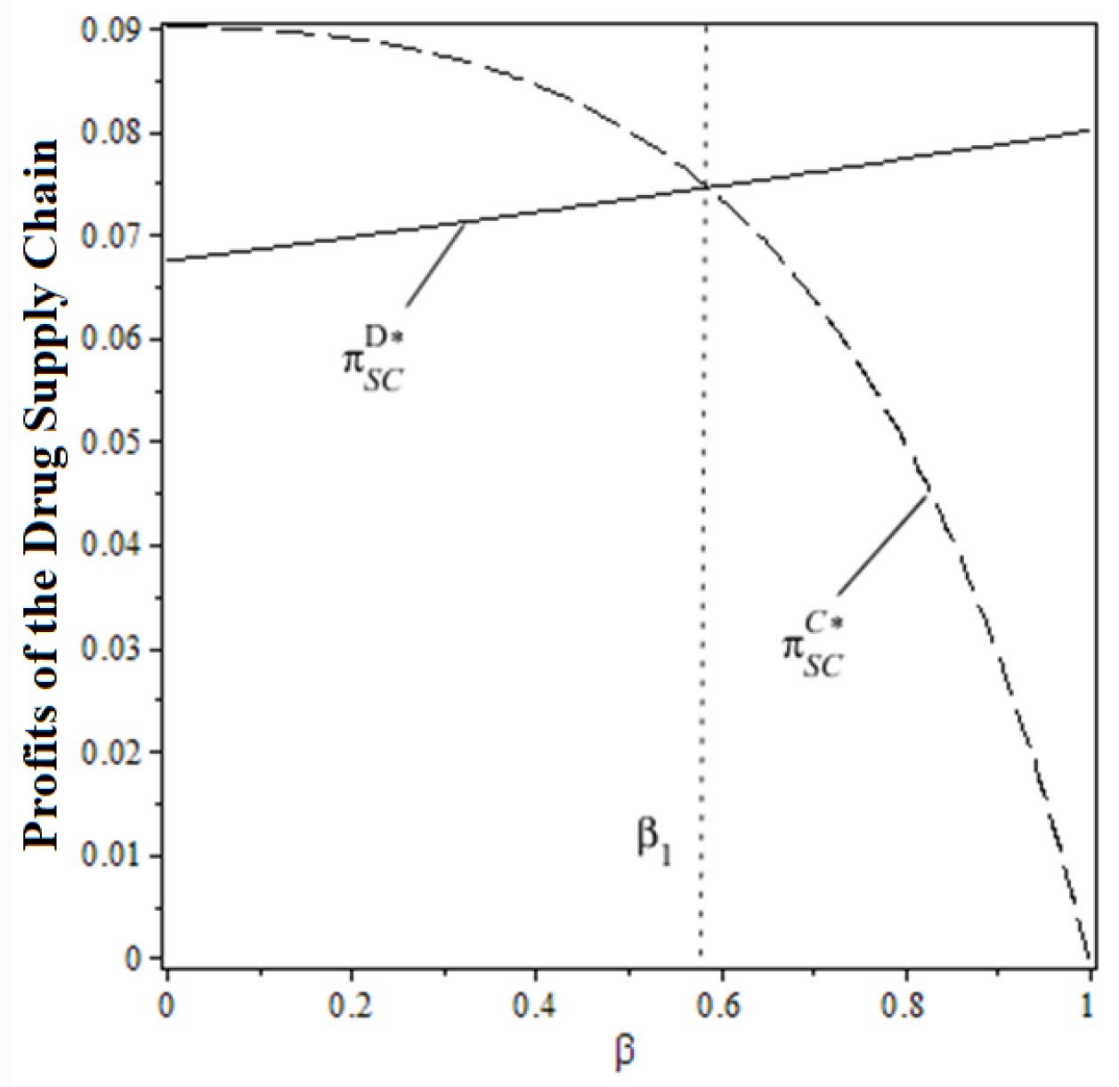
Profits of pharmaceutical supply chain under two decision-making scenarios.

**Figure 4:**
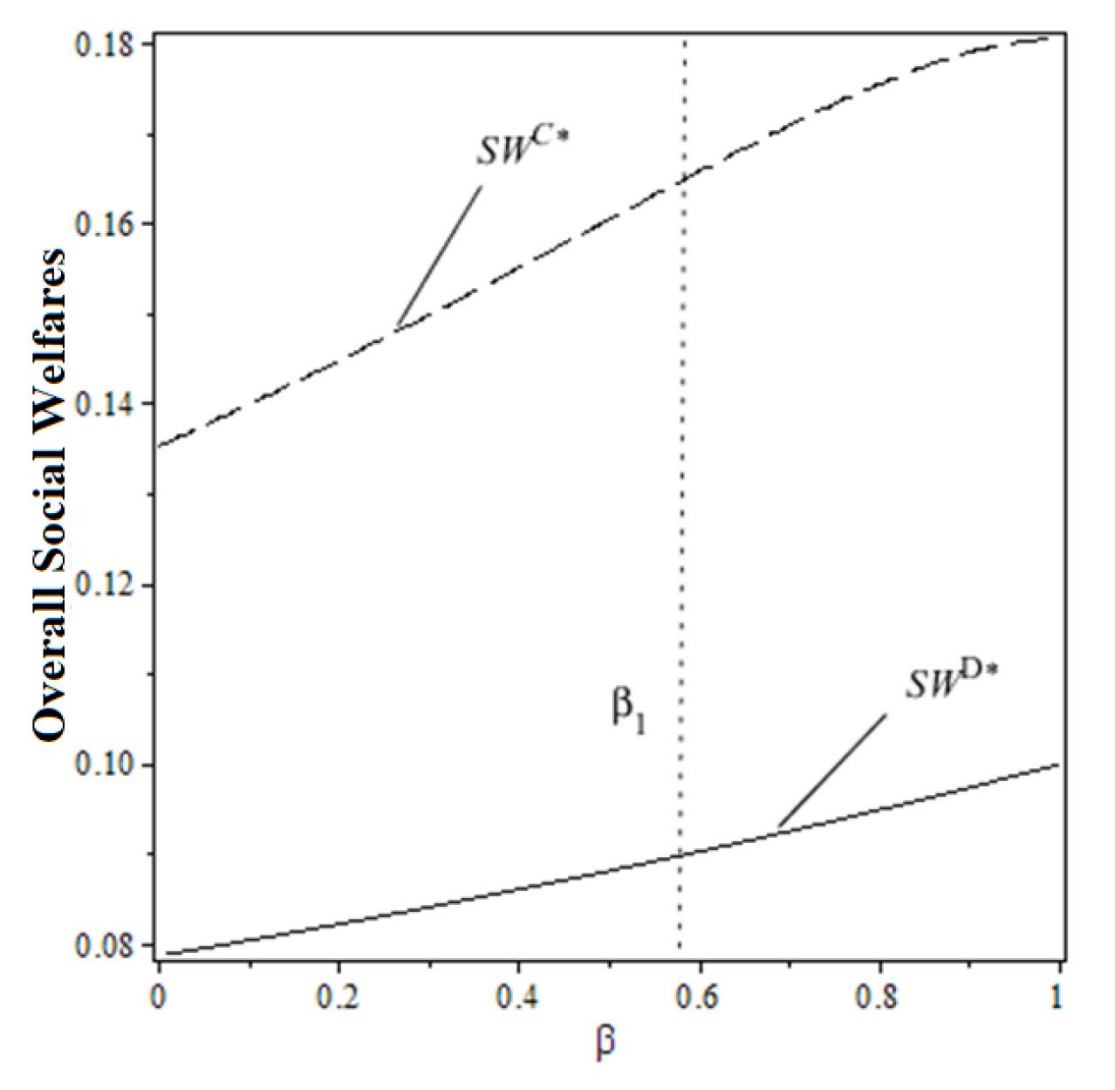
Overall social welfare under two decision-making scenarios.

From the perspective of social welfare, the overall social welfare under decentralized decision-making is always lower than the centralized one, regardless of the change in the public benefit of hospitals. To explain the impacts of the public benefit of hospitals on supply chain profit and social welfare under both decision-making scenarios, Figures 3, 4 are made based on $c_{h}=0.10$ , $c_{m}=0.30$ , η=0.05 .

From the figures, the individual rationality level of pharmaceutical supply chain members could affect the overall performance and social welfare under the decentralized decision-making scenario. To gain more benefits (profit or total utility), both drug suppliers and the public hospital have incentives to design a rational contract to achieve higher profits and social welfare, which the centralized decision-making scenario can obtain. In this case, designing an effective contract could be the solution.

## 5. Pharmaceutical supply chain coordination contract

When $0\leq \beta <\beta_{1}$ , the profit and social welfare of the pharmaceutical supply chain under centralized decision-making are greater than those under the decentralized one. An effective coordination contract could facilitate profit transfer within the pharmaceutical supply chain members to achieve more overall social welfare. When $\beta_{1}\leq \beta \leq 1$ , the profit of the pharmaceutical supply chain under centralized decision is smaller than the decentralized one, while the overall social welfare is larger than the decentralized one. It indicates that when the public benefit of hospitals is low, a proper coordination contract will not only result in higher profits for members of the pharmaceutical supply chain but will also generate greater social welfare. When the public benefit of hospitals is sufficiently high, none of the coordination contracts can make the pharmaceutical supply chain more profitable. In reality, when hospitals are expected to perform at a higher level of public benefit, they are often subsidized by the Government in China, for example, infectious disease hospitals, psychiatric hospitals, etc. These hospitals are fully funded by the government. This study focuses on how to design contracts to solve the issues in the pharmaceutical supply chain under the background of the zero-markup drug policy. The state-funded public hospitals are not the objectives of this study. Therefore, the new contract form in this study is only designed for the specific scenario of $0\leq \beta <\beta_{1}$ .

When $0\leq \beta <\beta_{1}$ , a “cost-sharing + fixed medical service fee + transfer payment” contract $\left(\theta ,\;a_{h}^{H},\;f\right)$ is designed for the cooperation between drug suppliers and public hospitals. θ refers to the proportion of the variable cost afforded by the drug suppliers to the overall cost afforded by the public hospitals. $a_{h}^{H}$ refers to the public hospital’s medical service fee for appointments made before the start of the sales season. f refers to the compensation to the public hospitals from the drug suppliers’ revenue according to the contracts after the end of the sales season. According to the coordination contract, the drug supplier shows their willingness to bear the variable cost of the medical service at the rate of $\theta \in \left[0,\;1\right]$ . Meanwhile, public hospitals must charge $a_{h}^{H}$ for the medical services. At the end of the selling period, the supplier promises to compensate the public hospital with a certain amount of revenue. In practice, drug suppliers often provide hospitals with diagnostic equipment to help with diagnosis and reduce medical treatment costs. Under this contract, the decision problem for the public hospital and the drug supplier can be expressed in the following equations:


(4) $$\max_{s}v_{h}^{H}=\left(a_{h}^{H}-c_{h}\right)\cdot q_{h}+\beta \cdot \overset{1}{\underset{\delta_{1}}{\int }}\left(\delta -p_{m}-a_{h}^{H}+\eta s\right)d\delta -\frac{1}{2}\left(1-\theta \right)s^{2}+f$$

(5) $$\max_{p_{m}}\pi_{m}^{H}=\left(p_{m}-c_{m}\right)\cdot q_{h}-\frac{1}{2}\theta s^{2}-f$$

Theorem 3:

Under the contract of “cost sharing + fixed medical service fees + transfer payments,” the equilibrium solution of the pharmaceutical supply chain is:


θ=1

$$a_{h}^{H}=\frac{2c_{h}-\eta^{2}c_{h}+\beta c_{m}-\beta }{2-\eta^{2}-\beta }$$

$$p_{m}^{H*}=\frac{1-\eta^{2}c_{m}-\beta c_{m}-c_{h}+c_{m}}{2-\eta^{2}-\beta }$$

$$p_{m}^{H*}+a^{H}=p^{C*}$$

$$s^{H*}=s^{C*}$$

The profits of public hospitals and drug suppliers are:


$$\pi_{h}^{H*}=-\frac{\beta {\left(1-c_{h}-c_{m}\right)}^{2}}{{\left(2-\eta^{2}-\beta \right)}^{2}}+f$$

$$\pi_{m}^{H*}=\frac{{\left(1-c_{h}-c_{m}\right)}^{2}\left(2-\eta^{2}\right)}{2{\left(2-\eta^{2}-\beta \right)}^{2}}-f$$

Theorem 3 shows that when the “cost-sharing + fixed medical service fee + transfer payment” contract between drug suppliers and public hospitals is enforced, the contract can trigger internal coordination within the pharmaceutical supply chain. The level of medical service that the patients received and the overall medical treatment fees (the sum of medical treatments and drugs) are the same as in the centralized decision-making scenarios. It means that patients get better service at lower prices, and the pharmaceutical supply chain gains greater profits under the “cost-sharing + fixed fee for medical services + transfer payments” contract. Due to θ=1 , the variable costs of medical services will be fully covered by the drug suppliers.

The pharmaceutical supply chain gains greater profit under the coordination contract mechanism, but this profit needs to be rationally distributed among the supply chain members to meet their respective participation constraints. To implement the contract more effectively, the participant constraints of the supply chain members are suggested to be $v_{h}^{H*}\geq v_{h}^{D*}$ , $\pi_{h}^{H*}\geq \pi_{h}^{D*}$ , and $\pi_{m}^{H*}\geq \pi_{m}^{D*}$ . Based on this constraint, Proposition 4 is obtained as follows:

Proposition 4:

When $0\leq \beta <\beta_{1}$ and $F_{2}\leq f\leq F_{1}$ , both the drug suppliers and the public hospitals are willing to accept contract $\left(\theta ,a_{h}^{H},f\right)$ . F_1_ and F_2_ can be calculated by the following equations:


(6) $$F_{1}=\frac{{\left(c_{h}+c_{m}-1\right)}^{2}\left(24-\eta^{6}-2\beta \eta^{4}-\beta^{2}\eta^{2}+8\eta^{4}+8\beta \eta^{2}-24\eta^{2}-8\beta \right)}{2{\left(\eta^{2}+\beta -2\right)}^{2}{\left(\eta^{2}+\beta -4\right)}^{2}}$$

(7) $$F_{2}=\frac{{\left(c_{h}+c_{m}-1\right)}^{2}\left(\begin{array}{l} 16-\eta^{6}-2\beta \eta^{4}-\beta^{2}\eta^{2}\\ +8\eta^{4}+4\beta \eta^{2}-4\beta^{2}-20\eta^{2}+8\beta \end{array}\right)}{2{\left(\eta^{2}+\beta -2\right)}^{2}{\left(\eta^{2}+\beta -4\right)}^{2}}$$

When the public benefit is lower and the transfer payment satisfies $f\in \left[F_{2},F_{1}\right]$ , the cooperation between the drug suppliers and the public hospitals could lead to a win-win profitable situation and achieve maximum overall social welfare. Moreover, a flexible profit distribution between the drug suppliers and public hospitals can be achieved by adjusting the transfer payment f .

**Figure 5:**
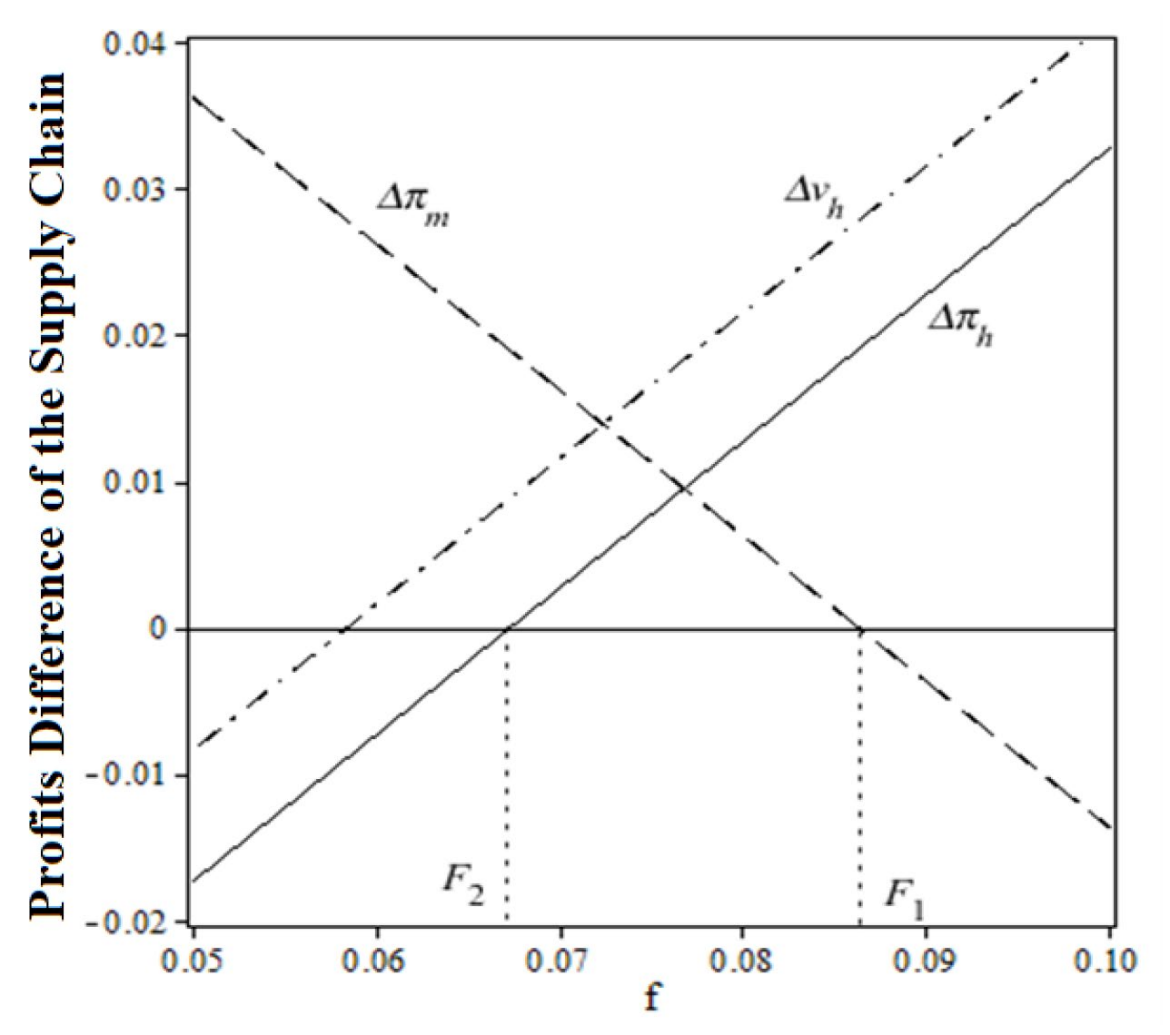
Relative profits difference and transfer payment f .

To prove the validity of the coordination parameter f , Figure 5 is drawn to show the relationship between the relative profits difference $\Delta \pi \left(\Delta v\right)$ and f at $c_{h}=0.1$ , $c_{m}=0.3$ , β=0.2 , η=0.05.

Before the coordination of the pharmaceutical supply chain, the relative profit (total utility) difference of public hospitals was a monotonically increasing function of a fixed payment f , while the relative profit of drug suppliers was a monotonically decreasing function of a fixed payment f . When the transfer payment satisfies $f\in \left[F_{2},F_{1}\right]$ , a win-win situation for all supply chain members can be obtained. Figure 5 also shows a certain flexibility of the fixed payment to induce a favorable distribution of the total profits among all the pharmaceutical supply chain members. In reality, f depends largely on the strength of the negotiating power between drug suppliers and hospitals. In China, public hospitals tend to have a greater negotiating advantage.

## 6. Conclusion

In the background of the drug zero-markups policy, this study innovatively builds a game theoretical model between drug suppliers and public hospitals. The influences of public benefit character on the medical service level, medical service fee, and drug prices are comprehensively analyzed. By comparing the profits and overall social welfare under decentralized decision-making and centralized decision-making in the pharmaceutical supply chain, the reasons for the lower performance of the pharmaceutical supply chain system under decentralized decision-making scenario are revealed. A coordination contract is designed to boost cooperation and facilitate the transfer of profits between different members of the pharmaceutical supply chain. Some conclusions are drawn as follows:

(1) In the background of the zero-markup drug policy, increasing the public benefit of hospitals can only reduce the price of medical services but not the price of drugs. The higher the public benefit of the hospital, the higher the level of medical services and the lower the overall fee for patients. Moreover, the increase in the public benefit of hospitals could improve both profits in the pharmaceutical supply chain and overall social welfare. From the perspective of profits, it is worth noting that simply increasing the public benefit of hospitals is advantageous to drug suppliers but will be resisted by hospitals.

(2) When the public benefit of a hospital is high, the profits of the pharmaceutical supply chain under the decentralized decision-making scenario are lower than those of the centralized one. When the public benefit of hospitals is low, the profits of the pharmaceutical supply chain under decentralized decision-making are higher than those of the centralized one. However, the social welfare of the pharmaceutical supply chain under the decentralized decision-making scenario is always lower than that of the centralized one, regardless of the public benefit of the hospital. It means that when the public benefit of the hospital is low, both drug suppliers and the public hospital have incentives to design a rational contract to achieve higher profits and social welfare. When the public benefit of the public hospital is high, the pharmaceutical supply chain cannot achieve a favorable state of internal profit distribution and coordination.

(3) When the public benefit of a hospital is low, the novel coordination contract of “cost sharing + fixed medical service fee + transfer payment” can coordinate the pharmaceutical supply chain to achieve a win-win profitable situation for all supply chain members and increase the patient surplus number. When the public benefit of hospitals is high, the pharmaceutical supply chain needs to receive external subsidies to achieve higher social welfare, such as government subsidies and donations from charitable organizations.

## Data availability statement

The original contributions presented in the study are included in the article/Supplementary material, further inquiries can be directed to the corresponding author.

## Author contributions

NZ and SL contributed to the study design and wrote the manuscript drafts. GZ and CL supervised the study and provided suggestions for the revision of the manuscript drafts. SL, GZ, CL, and NY provided some reviews on the manuscript. All authors contributed to the article and approved the submitted version.

## References

[1] Xiong W Deng Y Yang Y Zhang Y Pan J. Assessment of medical service pricing in China’s healthcare system: challenges, constraints, and policy recommendations. Front Public Health. (2021) 9:1932. doi: 10.3389/fpubh.2021.787865 PMC8661692 34900924

[2] Zhang X Lai H Zhang L He J Fu B Jin C. The impacts and unintended consequences of the nationwide pricing reform for drugs and medical services in the urban public hospitals in China. BMC Health Serv Res. (2020) 20:1058–12. doi: 10.1186/s12913-020-05849-4, PMID: 33225941 PMC7682084

[3] Ramesh M Wu X He AJ. Health governance and healthcare reforms in China. Health Policy Plan. (2014) 29:663–72. doi: 10.1093/heapol/czs109, PMID: 23293100

[4] He Z Zhou J. Can zero-markup policy for drug sales in public hospitals resolve the problem of ‘seeing a doctor is too expensive’ in China? A case study of four municipal general tertiary hospitals at H City in Z Province. J Chinese Governance. (2017) 2:329–42. doi: 10.1080/23812346.2017.1342899

[5] Wang Y Zhang Y Ma C Jiang Y Li Y Wang X. Limited effects of the comprehensive pricing healthcare reform in China. Public Health. (2019) 175:4–7. doi: 10.1016/j.puhe.2019.06.014, PMID: 31369975

[6] Shi XF Zhu DW Man XW Wang W Zhu K Nicholas S. “The biggest reform to China’s health system”: did the zero-markup drug policy achieve its goal at traditional Chinese medicines county hospitals? Health Policy Plan. (2019) 34:483–91. doi: 10.1093/heapol/czz053, PMID: 31363744

[7] Zhou ZL Su YF Campbell B Zhou Z Gao J Yu Q. The impact of China’s zero-markup drug policy on county hospital revenue and government subsidy levels. J Asian Public Policy. (2015) 8:102–16. doi: 10.1080/17516234.2015.1005561

[8] Yi H Miller G Zhang L Li S Rozelle S. Intended and unintended consequences of China’s zero markup drug policy. Health Aff. (2015) 34:1391–8. doi: 10.1377/hlthaff.2014.1114, PMID: 26240254

[9] Qiu T Hanna E Ma F Toumi M. The “second negotiation” policy in Chinese hospitals. Value Health. (2018) 21:S44–5. doi: 10.1016/j.jval.2018.07.341

[10] Zang W Zhou M Zhao S. Deregulation and pricing of medical services: a policy experiment based in China. BMC Health Serv Res. (2021) 21:1–10. doi: 10.1186/s12913-021-06525-x 34034722 PMC8146237

[11] Wang Y Yang L Sun Q Wang M Huang S Ming X. Research on the medical service pricing policy in China’s ongoing healthcare reform. Chinese J Hosp Administrat. (2017) 33:641–644. doi: 10.3760/cma.j.issn.1000-6672.2017.09.001

[12] Wang H Jin C Wang W Gong L He Y Peng Y. Development of price comparison method system for Shanghai's medical service pricing. Chinese J Hosp Administrat. (2015) 31:627–630. doi: 10.3760/cma.j.issn.1000-6672.2015.08.021

[13] Świderska GK Raulinajtys-Grzybek M. Cost-based pricing of healthcare services. Relevance of a resource-based costing system. ZTR. (2013) 2013:117–37. doi: 10.5604/16414381.1063616

[14] Prieto-Pinto L Garzón-Orjuela N Lasalvia P Castañeda-Cardona C Rosselli D. International experience in therapeutic value and value-based pricing: a rapid review of the literature. Value Health Reg Issues. (2020) 23:37–48. doi: 10.1016/j.vhri.2019.11.008, PMID: 32688214

[15] Grennan M. Bargaining ability and competitive advantage: empirical evidence from medical devices. Manag Sci. (2014) 60:3011–25. doi: 10.1287/mnsc.2014.2006

[16] Duan J Lin Z Jiao F. A game model for medical service pricing in DRGs system. Front Public Health. (2021) 9:737788. doi: 10.3389/fpubh.2021.737788 34917572 PMC8669393

[17] Allen R Gertler P. Regulation and the provision of quality to heterogenous consumers: the case of prospective pricing of medical services. J Regul Econ. (1991) 3:361–75. doi: 10.1007/BF00138477

[18] Robinson J. Hospitals respond to Medicare payment shortfalls by both shifting costs and cutting them, based on market concentration. Health Aff. (2011) 30:1265–71. doi: 10.1377/hlthaff.2011.0220, PMID: 21734199

[19] di Giacomo M Piacenza M Siciliani L Turati G. Do public hospitals respond to changes inDRGprice regulation? The case of birth deliveries in theItalianNHS. Health Econ. (2017) 26:23–37. doi: 10.1002/hec.3541, PMID: 28940919

[20] Roberts ET Chernew ME McWilliams JM. Market share matters: evidence of insurer and provider bargaining over prices. Health Aff. (2017) 36:141–8. doi: 10.1377/hlthaff.2016.0479, PMID: 28069857

[21] Hsiao WC Cheng SH Yip W. What can be achieved with a single-payer NHI system: the case of Taiwan. Soc Sci Med. (2019) 233:265–71. doi: 10.1016/j.socscimed.2016.12.006, PMID: 29054594

[22] Sheaff R Morando V Chambers N Exworthy M Mahon A Byng R. Managerial workarounds in three European DRG systems. J Health Organ Manag. (2020) 34:295–311. doi: 10.1108/JHOM-10-2019-0295, PMID: 32364346 PMC7406989

[23] Wang JJ Li ZP Shi JJ Chang AC(J). Hospital referral and capacity strategies in the two-tier healthcare systems. Omega. (2021) 100:102229. doi: 10.1016/j.omega.2020.102229

[24] Li B Hou PW Chen P Li QH. Pricing strategy and coordination in a dual channel supply chain with a risk-averse retailer. Int J Prod Econ. (2016) 178:154–68. doi: 10.1016/j.ijpe.2016.05.010

[25] Vosooghidizaji M Taghipour A Canel-Depitre B. Supply chain coordination under information asymmetry: a review. Int J Prod Res. (2020) 58:1805–34. doi: 10.1080/00207543.2019.1685702

[26] Xu X Zhang M He P. Coordination of a supply chain with online platform considering delivery time decision. Trans Res E Logist Trans Rev. (2020) 141:101990. doi: 10.1016/j.tre.2020.101990

[27] Zhao T Xu X Chen Y Liang L Yu Y Wang K. Coordination of a fashion supply chain with demand disruptions. Trans Res E Logist Trans Rev. (2020) 134:101838. doi: 10.1016/j.tre.2020.101838

[28] Panda S Modak N Basu M Goyal SK. Channel coordination and profit distribution in a social responsible three-layer supply chain. Int J Prod Econ. (2015) 168:224–33. doi: 10.1016/j.ijpe.2015.07.001

[29] Xu L Wang C Zhao J. Decision and coordination in the dual-channel supply chain considering cap-and-trade regulation. J Clean Prod. (2018) 197:551–61. doi: 10.1016/j.jclepro.2018.06.209

[30] Biswas I Gupta R Tiwari S Talluri S. Multi-echelon supply chain coordination: contract sequence and cut-off policies. Int J Prod Econ. (2023) 259:108823. doi: 10.1016/j.ijpe.2023.108823

[31] Gao J Xiao Z Wei H. Competition and coordination in a dual-channel green supply chain with an eco-label policy. Comput Ind Eng. (2021) 153:107057. doi: 10.1016/j.cie.2020.107057

[32] Moon I Jeong YJ Saha S. Investment and coordination decisions in a supply chain of fresh agricultural products. Oper Res. (2020) 20:2307–31. doi: 10.1007/s12351-018-0411-4

[33] Heydari J Govindan K Sadeghi R. Reverse supply chain coordination under stochastic remanufacturing capacity. Int J Prod Econ. (2018) 202:1–11. doi: 10.1016/j.ijpe.2018.04.024

[34] Weraikat D Zanjani MK Lehoux N. Two-echelon pharmaceutical reverse supply chain coordination with customers incentives. Int J Prod Econ. (2016) 176:41–52. doi: 10.1016/j.ijpe.2016.03.003

[35] Nematollahi M Hosseini-Motlagh SM Ignatius J Goh M Nia MS. Coordinating a socially responsible pharmaceutical supply chain under periodic review replenishment policies. J Clean Prod. (2018) 172:2876–91. doi: 10.1016/j.jclepro.2017.11.126

[36] Tat R Heydari J Rabbani M. Corporate social responsibility in the pharmaceutical supply chain: an optimized medicine donation scheme. Comput Ind Eng. (2021) 152:107022. doi: 10.1016/j.cie.2020.107022

[37] Brekke KR Siciliani L Straume OR. Competition and waiting times in hospital markets. J Public Econ. (2008) 92:1607–28. doi: 10.1016/j.jpubeco.2008.02.003

[38] Eggleston K Yip W. Hospital competition under regulated prices: application to urban health sector reforms in China. Int J Health Care Finance Econ. (2004) 4:343–68. doi: 10.1023/B:IHFE.0000043762.33274.4f 15467409

