# Supplementary material for: Medical service pricing and pharmaceutical supply chain coordination contracts under the zero-markup drug policy
Source: Front Public Health. 2023 Oct 19;11:1208994. doi: 10.3389/fpubh.2023.1208994 (PMC10621740; doi:10.3389/fpubh.2023.1208994)
Supplement: Supplementary file 1 [file Data_Sheet_1.docx]

Appendix A: Proofs

**Proof of Theorem 1:**

Given $a_{h}^{D}$ and $s^{D}$, solving the reaction function of the drug supplier of formula (2) can be obtained: $p_{m}^{D*}=1/2 \eta s-1/2 a_{h}+1/2 c_{m}+1/2$.

Substitute $p_{m}^{D*}$ into equation (1), the Hessian matrix of the equation (1) is:

$H=\left(\begin{matrix} \frac{\partial^{2}v_{h}^{D}}{\partial s^{2}},\frac{\partial^{2}v_{h}^{D}}{\partial a_{h}\partial s} \\ \frac{\partial^{2}v_{h}^{D}}{\partial s\partial a_{h}},\frac{\partial^{2}v_{h}^{D}}{\partial a_{h}^{2}} \end{matrix}\right)\Rightarrow\left(\begin{matrix} \frac{1}{4} \beta-1,\frac{1}{2}\eta-\frac{1}{4} \beta\eta\\ \frac{1}{2}\eta-\frac{1}{4} \beta\eta\text{,}\frac{1}{4} \beta\eta^{2}-1 \end{matrix}\right)$. According to the Hessian matrix determination method, when $\frac{\partial^{2}v_{h}^{D}}{\partial s^{2}}<0$and$\frac{\partial^{2}v_{h}^{D}}{\partial s^{2}}\cdot\frac{\partial^{2}v_{h}^{D}}{\partial a_{h}^{2}}-(\frac{\partial^{2}v_{h}^{D}}{\partial a_{h}\partial s})^{2}>0$, the Hessian matrix is negative definite and the equation (1) has a maximum value. In this equation, since$\beta\in\left[0,1\right]$and$\eta\in\left(0,1\right)$,$\frac{\partial^{2}v_{h}^{D}}{\partial s^{2}}=\frac{1}{4} \beta-1<0$;

$\frac{\partial^{2}v_{h}^{D}}{\partial s^{2}}*\frac{\partial^{2}v_{h}^{D}}{\partial a_{h}^{2}}-(\frac{\partial^{2}v_{h}^{D}}{\partial a_{h}\partial s})^{2}=\left(\frac{1}{4} \beta-1\right)\cdot\left(\frac{1}{4} \beta\eta^{2}-1\right)-\left(\frac{1}{2}\eta-\frac{1}{4} \beta\eta\right)^{2}=1-\frac{1}{4} \beta-\frac{1}{4} \eta^{2}>0$, then the objective function Hessian matrix is negative definite, and there is a unique optimal solution.

This can be obtained: $a_{h}^{D*}=\frac{c_{h}\left(2-\eta^{2}\right)+\left(1-c_{m}\right)\left(2-\beta\right)}{4-\eta^{2}-\beta}$，$s^{D*}=\frac{\eta\left(1-c_{h}-c_{m}\right)}{4-\eta^{2}-\beta}$.

Plugging $a_{h}^{D*}$ and $s^{D*}$ back into $p_{m}^{D*}$, we have: $p_{m}^{D*}=\frac{1-\eta^{2}c_{m}-\beta c_{m}-c_{h}+3 c_{m}}{4-\eta^{2}-\beta}$.

The above equilibrium decisions are substituted into equations (1) and (2) to obtain the profit and utility.

**Proof of Proposition 1:**

It is easy to obtain the derivative of equilibrium results under decentralized decision-making.

**Proof of Theorem 2:**

The Hessian matrix of the equation (3) is: $H=\left(\begin{matrix} \frac{\partial^{2}v^{C}}{\partial^{2}p^{C}},\frac{\partial^{2}v_{h}^{C}}{\partial p^{C}\partial s^{C}} \\ \frac{\partial^{2}v_{h}^{C}}{\partial p^{C}\partial s^{C}},\frac{\partial^{2}v_{h}^{D}}{\partial^{2}s^{C}} \end{matrix}\right)\Rightarrow\left(\begin{matrix} \beta-2,\eta- \beta\eta\\ \eta-\beta\eta\text{,}\beta\eta^{2}-1 \end{matrix}\right)$. Similar to the proof of the equation (1), Because$\beta-2<0$and$\left(\beta-2\right)\left(\beta\eta^{2}-1\right)-\left(\eta-\beta\eta\right)^{2}=-\eta^{2}-\beta+2>0$, the objective function Hessian matrix is negative, the function (3) has a maximum value.

This can be obtained: $p^{C*}=\frac{\left(1-\eta^{2}\right)\left(c_{h}+c_{m}\right)-\beta+1}{2-\eta^{2}-\beta}$, $s^{C*}=\frac{\eta\left(1-c_{h}-c_{m}\right)}{2-\eta^{2}-\beta}$.

The above equilibrium decisions are substituted into equation (3) to obtain the profit and utility.

**Proof of Proposition 2:**

It is easy to obtain the derivative of equilibrium results under centralized decision-making.

**Proof of Proposition 3:**

1. $s^{D*}-s^{C*}\text{=} -\frac{2\eta\left(1-c_{h}-c_{m}\right)}{\left(4-\eta^{2}-\beta\right)\left(2-\eta^{2}-\beta\right)}<0$. Similarly, we have $p_{m}^{D*}+a_{h}^{D*}>p^{C*}$.
2. Let $\Delta\pi_{SC}=\pi_{SC}^{D*}-\pi_{SC}^{C*}$, we have $\Delta\pi_{SC}\text{=}\frac{2\left(c_{h}+c_{m}-1\right)^{2}\left(-\beta\eta^{2}-\beta^{2}+\eta^{2}+4 \beta-2\right)}{\left(\eta^{2}+\beta-4\right)^{2}\left(\eta^{2}+\beta-2\right)^{2}}$. By observing the equation it is clear that$\left(-\beta\eta^{2}-\beta^{2}+\eta^{2}+4 \beta-2\right)$will determine the positive and negative of $\Delta\pi_{SC}$. Let $Y=-\beta\eta^{2}-\beta^{2}+\eta^{2}+4 \beta-2$, We know that $\beta$has two roots: $\beta_{1}=2-\frac{1}{2} \eta^{2}-\frac{1}{2}\sqrt{\eta^{4}-4 \eta^{2}+8}$, $\beta_{2}=2-\frac{1}{2} \eta^{2}+\frac{1}{2}\sqrt{\eta^{4}-4 \eta^{2}+8}$. Because $\beta\in\left[0,1\right]$, We choose $\beta_{1}$. When $0\leq\beta<\beta_{1}$, $\Delta\pi_{SC}<0$. When $\beta_{1}<\beta\leq1$, $\Delta\pi_{SC}>0$.

$SW^{D*}-SW^{C*}\text{=}- \frac{2\left(c_{h}+c_{m}-1\right)^{2}\left(\beta\eta^{2}+\beta^{2}-2 \eta^{2}-5 \beta+5\right)}{\left(\eta^{2}+\beta-4\right)^{2}\left(\eta^{2}+\beta-2\right)^{2}}$, when $\beta\in\left[0,1\right]$, there is always $SW^{D*}<SW^{C*}$.

**Proof of Theorem 3:**

Solving the reaction function$p_{m}^{H*}$of the drug supplier of formula (5) can be obtained: $p_{m}^{H*}=1/2 \eta s-1/2 a_{h}+1/2 c_{m}+1/2$.

Substitute $p_{m}^{H*}$ into equation (4), the optimal value of the level of medical care can be obtained: $s^{H*}=\frac{\eta\left(\beta a_{h}+\beta c_{m}-\beta-2 a_{h}+2 c_{h}\right)}{\beta\eta^{2}+4 \theta-4}$.

Plugging $s^{H*}$ back into $p_{m}^{H*}$, we have: $p_{m}^{H*}=\frac{\eta^{2} \left(\beta a_{h}+\beta c_{m}-\beta-2 a_{h}+2 c_{h}\right)}{2\left(\beta\eta^{2}+4 \theta-4\right)}-\frac{1}{2} a_{h}+\frac{1}{2} c_{m}+\frac{1}{2}$.

Let $s^{H*}\text{=}s^{C*}$,$a_{h}^{H}\text{+}p_{m}^{H*}\text{=}p^{C*}$, and solve the system of equations to obtain:$\theta=1$, $a_{h}^{H}=\frac{2 c_{h}-\eta^{2}c_{h}+\beta c_{m}-\beta}{2-\eta^{2}-\beta}$.

The above equilibrium decisions are substituted into equations (4) and (5) to obtain the profit and utility.

**Proof of Proposition 4:**

When $0<\beta<\beta_{1}$, The conditions for the pharmaceutical supply chain to provide compensation strategies are:$\pi_{m}^{H*}\geq\pi_{m}^{D*}$.

Plug it in and we get:$f\leq\frac{\left(c_{h}+c_{m}-1\right)^{2}\left(16-\eta^{6}-2 \beta\eta^{4}-\beta^{2}\eta^{2}+8 \eta^{4}+4 \beta\eta^{2}-4 \beta^{2}-20 \eta^{2}+8 \beta\right)}{2\left(\eta^{2}+\beta-2\right)^{2}\left(\eta^{2}+\beta-4\right)^{2}}=F_{1}$.

When $0<\beta<\beta_{1}$, the conditions for medical institutions to accept the compensation strategy are:$\pi_{h}^{H*}\geq\pi_{h}^{D*}$，$v_{h}^{H*}\geq v_{h}^{D*}$.

Plug them in and we get: $f\geq\frac{\left(c_{h}+c_{m}-1\right)^{2}\left(24-\eta^{6}-2 \beta\eta^{4}-\beta^{2}\eta^{2}+8 \eta^{4}+8 \beta\eta^{2}-24 \eta^{2}-8 \beta\right)}{2\left(\eta^{2}+\beta-2\right)^{2}\left(\eta^{2}+\beta-4\right)^{2}}=F_{2}$.
